# Interactions between the Physical and Social Environments with Adverse Pregnancy Events Related to Placental Disorders—A Scoping Review

**DOI:** 10.3390/ijerph17155421

**Published:** 2020-07-28

**Authors:** Yolisa Prudence Dube, Newton Nyapwere, Laura A. Magee, Marianne Vidler, Sophie E. Moore, Benjamin Barratt, Rachel Craik, Peter von Dadelszen, Prestige Tatenda Makanga, The PRECISE Network

**Affiliations:** 1 Department of Surveying and Geomatics, Midlands State University, P. Bag 9055, Gweru, Zimbabwe; nyapweren@staff.msu.ac.zw (N.N.); makangap@staff.msu.ac.zw (P.T.M.); 2 Department of Women and Children’s Health, School of Life Course Sciences, Faculty of Life Sciences and Medicine, King’s College London, Strand, London WC2R 2LS, UK; laura.a.magee@kcl.ac.uk (L.A.M.); sophie.moore@kcl.ac.uk (S.E.M.); rachel.craik@kcl.ac.uk (R.C.); PVD@kcl.ac.uk (P.v.D.); 3 Department of Obstetrics and Gynaecology and BC Children’s Hospital Research Institute, University of British Columbia, Suite 930, 1125 Howe Street, Vancouver, BC V6Z 2K8, Canada; Marianne.Vidler@cw.bc.ca; 4 Medical Research Council (MRC) Unit, LSHTM Atlantic Boulevard, Fajara P. O. Box 273, Banjul, The Gambia; 5 Environmental Research Group, MRC Centre for Environment and Health, Imperial College London, Michael Uren Biomedical Engineering Hub, White City Campus, Wood Lane, London W12 0BZ, UK; b.barratt@imperial.ac.uk; 6 NIHR HPRU in Environmental Exposures and Health, Imperial College London, Michael Uren Biomedical Engineering Hub, White City Campus, Wood Lane, London W12 0BZ, UK

**Keywords:** placental function, environment, social determinants, Africa

## Abstract

Background: Due to different social and physical environments across Africa, understanding how these environments differ in interacting with placental disorders will play an important role in developing effective interventions. Methods: A scoping review was conducted, to identify current knowledge on interactions between the physical and social environment and the incidence of placental disease in Africa. Results: Heavy metals were said to be harmful when environmental concentrations are beyond critical limits. Education level, maternal age, attendance of antenatal care and parity were the most investigated social determinants. Conclusions: More evidence is needed to determine the relationships between the environment and placental function in Africa. The results show that understanding the nature of the relationship between social determinants of health (SDH) and placental health outcomes plays a pivotal role in understanding the risk in the heterogenous communities in Africa.

## 1. Introduction

Complications arising from the ischaemic placental disorders (i.e., pre-eclampsia, intrauterine growth restriction [IUGR], and placental abruption) [1] contribute to the high maternal and perinatal mortality and morbidity rates, particularly stillbirth [2], observed in sub-Saharan Africa. About 75% of the global burden of stillbirths occurs in sub-Saharan Africa and south Asia, with 60% of these deaths occurring in rural areas [2]. These statistics speak to disparities in the incidence of placental disorders within and across countries and regions. These disparities are evidence of potentially preventable contextual social and environmental factors that may contribute to these adverse events. Health inequities and inequalities disproportionately contribute to preventable morbidities and deaths [3].

Many organisations have taken the initiative to tackle the issue of social determinants of health (SDH) as a way of improving health. The emphasis is on creating healthy social and physical environments for all. The neighbourhood and built environment, social and community context and an individual’s education and stability are some of the key areas of SDHs [4]. Social inclusion/exclusion is one of the SDHs that lead to health inequalities. It results from a combination of factors including occupational status, gender, religion, income, education, cultural and gender norms and employment status, as examined by the Social Exclusion Knowledge Network (SEKN) [5]. In addition, models developed by the Employment Conditions Knowledge Network have shown that the employment status and sector are socioeconomic factors that affect not only the health of the worker but that of the family [6]. A study has shown how changes in socio-economic inequalities in post-apartheid South Africa have affected changes in health inequalities [7]. Due to different cultural, political and physical environments across Africa, understanding how these environments differ in interacting with placental disorders will play an important role in developing effective interventions. Currently, there is not enough evidence to determine and compare how these interactions differ across Africa.

Tackling the SDHs may be important in preventing adverse health events related to placental disorders. To develop targeting interventions to reduce the impact of placental disorders, it is essential to identify the social, economic and physical determinants of maternal health, specifically placental function, and understand how the relationships may vary across social groups within and across countries in Africa. It is now a global health policy priority to implement policies and programmes responsive to socio-cultural, contextual, regional and national societal heterogeneity in Africa [8,9,10,11]. There is a need to first understand the social structure and physical environments of different social groups to implement targeted policies and programmes.

A scoping review approach is considered when the objective is to identify existing evidence in a specific field, clarify key concepts in the literature, examine how research is conducted on a specific topic, identify key factors related to a concept, identify and analyse gaps in the knowledge base and as a precursor to a systematic review [12]. Our aims were to identify current knowledge on interactions between the physical and social environment and the risk of placental disease in Africa, and to identify current knowledge gaps. In this scoping review, the physical environmental factors were defined as harmful substances (e.g., air, water and land pollution), the built environment and climatic conditions. The social environment included the socioeconomic characteristics (e.g., occupation, education, income, place of residence), sociodemographic characteristics (e.g., age, ethnicity, race) and sociocultural characteristics (e.g., religion, health care seeking behaviour).

## 2. Materials and Methods 

The York methodology of Arksey and O’Malley, 2005, [13] was adopted as our intended aim was to provide a broader perspective on the existing literature surrounding the interactions between the environmental and socio-geographical factors related to placental health outcomes; these are low birth weight, pre-eclampsia, stillbirth, foetal growth restriction, IUGR and preterm birth. Methodological quality assessment was not considered on inclusion/exclusion based on quality scores. The review followed the steps highlighted by Arksey and O’Malley:

Stage 1. Identifying the research question
Stage 2. Identifying relevant studies
Stage 3. Study selection
Stage 4. Charting the data
Stage 5. Collating, summarising and reporting the results.

Table 1 lists the key themes and keywords used to search for relevant literature on Google and Google Scholar. The returned articles were used to further build more relevant search terms by scanning through them to identify key terms used, which were then added to the search terms. The search terms were grouped into three themes and a search using all the identified search terms was then undertaken across all included databases. The databases searched were Web of Science, EBSCOhost and PubMed. Studies carried out in Africa and published up to the day of the search (i.e., 2 February 2019), in English, were considered for inclusion in this search. The bibliographies were saved to Zotero.

A sample of the themes and search terms used is provided in Table 1.

Three authors independently reviewed abstracts and full articles. The final set of papers that met the criteria for the review were determined by consensus.

## 3. Results

**Figure 1:**
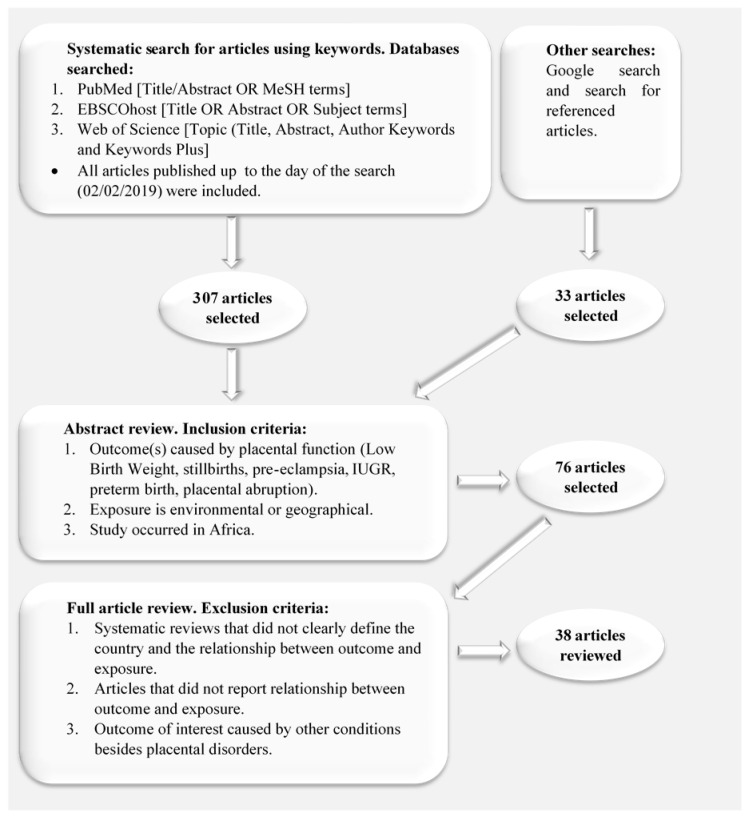
Diagrammatic illustration of the methodology adopted in selecting relevant articles for this scoping review write-up.

The literature search and the subsequent abstract and full article review yielded 38 articles that met the inclusion criteria, whose distribution is shown in Figure 1. Most of the included studies were retrospective, making use of patient records following discharge from, mostly referral, health facilities. 

**Figure 2:**
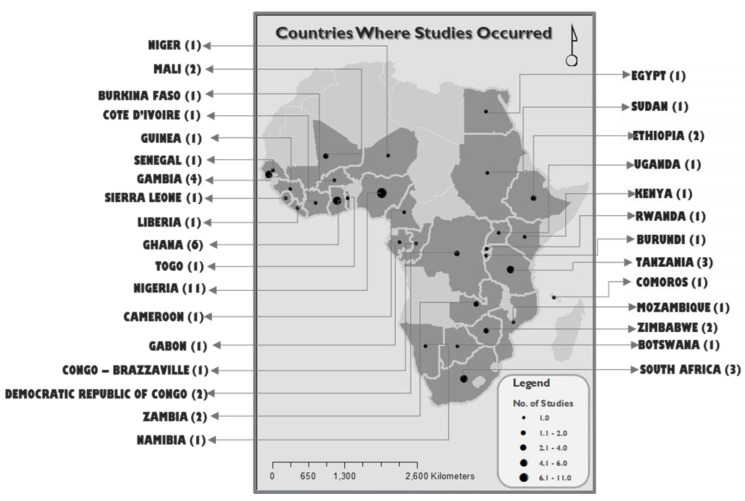
Distribution and number of research articles per country.

Figure 2 shows the countries where reviewed studies occurred and the number of articles from those countries. All the articles included involved five different study designs. These included 14 cohort studies, 11 case-control studies, seven cross-sectional studies, three randomised controlled studies, one prospective descriptive study and two review papers. A total of eight articles investigated the effect of the physical environment, usually discussed accordingly where women live and how this may have led to adverse pregnancy events. The crude associations were considered in reporting the relationships between the exposures and outcomes investigated in the reviewed articles. These articles focused on environmental toxicants, particularly heavy metals, environmental tobacco smoke (ETS) and household and ambient air pollution. Heavy metals discussed in the review articles were lead (Pb), arsenic (As) and cadmium (Cd), which were said to be harmful to the mother and the infant when environmental concentrations are beyond critical limits. Education level, maternal age, attendance of antenatal care and parity were the most investigated social determinants.

### 3.1. Social and Demographic Factors (Table 2)

A total of 30 articles discussed the interactions between socioeconomic and demographic factors and placental disorders. Education level, parity and maternal age were the most investigated factors (Table 2).

Education level was found to be associated with stillbirths, low birth weight (LBW) and pre-eclampsia [14,15,16,17,18,19,20,21,22,23,24,25,26]. The main reason for the protective effect of high education level was the mother’s knowledge of potential effects of not seeking antenatal care. Some of the studies that showed no association between education level and placental disorders suggested that the mother may be educated but the health attendants may not have enough knowledge to detect complications resulting from placental disorders. Maternal employment status is associated with LBW and stillbirth [15,20,21,27,28,29,30]. Maternal unemployment [28,29] and labour work [30] increased the risk of LBW and stillbirths, whereas maternal employment was protective [15,21,27]. In some studies, occupation of the women could not be clearly defined as there could be combinations of occupations, for instance a housewife being unemployed, an entrepreneur or a peasant [18]. 

Parity was found to be associated with stillbirths, abruptio placentae, LBW, placental abnormalities, hypertension and eclampsia [14,16,20,21,22,27,31,32,33,34,35]. High parity presents as a risk factor due to the characteristics of multiparous women, which include high tendencies of performing unsupervised vaginal deliveries, not seeking antenatal care and their advanced age [22]. Grand multiparity is also associated with poor health services and low socioeconomic status [36]. The effect of parity changes with different types of stillbirths. Maternal age is associated with stillbirth, LBW and pre-eclampsia. Adolescents were found to be at higher risk of LBW children due to not knowing the importance of self-care and childbearing and the steps to take in maintaining their pregnancies [21]. The same reason is true for primiparous women as these are often adolescents [34]. Grand multiparous and older women tend to become overconfident due to previous successful births, thus, they do not book for care at a health facility [20]. Grand multiparity could be an indicator for, or cause of, poor nutrition because of depleting nutritional stores resulting from multiple pregnancies and long periods of breast feeding [37].

Antenatal care (ANC) booking status is associated with stillbirths and placenta previa [20,22]. Not booking increases the risk of stillbirths and placenta praevia [20,22]. Although quality ANC does not prevent placenta praevia, it reduces the adverse outcomes associated with it. Women who were booked had lower prevalence of placental function related complications because these complications were detected early through screening [22]. The authors recommended the use of interventions that create awareness of placenta praevia and those that help in managing the outcomes associated with placenta previa will ensure early detection of placenta previa and aversion of adverse outcomes resulting from by placenta previa. Prenatal care-seeking behaviour is associated with the detection of pre-eclampsia and LBW [16,18,19,31,34]. The high prevalence of intrapartum-related stillbirths, especially in rural areas, has been attributed to late diagnosis of the complications by the traditional birth attendants and unskilled birth attendants who are not equipped with enough knowledge to diagnose such complications [20,22]. Poor economic status also attributes to the low utilisation of health care services by pregnant women [20].

The husband’s employment status, maternal birth season, paternal birth season, diet, type of union, maternal employment sector, socioeconomic status, season of birth, unstable income, unplanned pregnancy, place of delivery and wealth index are all socio-geographical factors associated with LBW [15,21,38,39,40,41]. All these factors were only investigated for their association with LBW. The high prevalence of LBW children born to mothers who are peasants was attributed to the strenuous field work they were subjected to during pregnancy [18,30]. The vine and root vegetable diet was found to be protective, possibly due to the low levels of cadmium in the soil the mothers grew their vegetables in, in the coastal geographical area of Western Cape Province of South Africa, which was the study area [39]. Residency status is associated with LBW [15,19]. Women who lived in rural areas were at higher risk of adverse outcomes compared to those living in urban areas, likely due to poor socioeconomic status, labour on the farms and poor use of health care services [15,19].

Maternal nutrition and drinking and smoking were investigated for their association with preterm birth [19]. Poor nutrition, which has been attributed to LBW, is associated with lower socioeconomic status [21]. The research showed that the season of birth of the mother has a greater effect on the infant’s birth weight than the nourishment of the offspring itself [38]. In a Zimbabwean study, it was found that a majority of women in the study drank mahewu (a traditional Southern African non-alcoholic drink made from fermented mealie-meal), which appeared to provide some protection against preterm births [19]. Adolescents were found to be at higher risk of having LBW infants and it is suggested that this is because they are still growing; hence, nutrients are shared between them and the foetus [34]. However, this is unlikely as pregnancy causes adolescents’ epiphyses to fuse, thereby ending growth.

In one study from Dakar, Senegal, it was observed that the use of skin lightening cream is associated with LBW and placental weight [42]. Our interest in the use of skin lightening cream was more social than biological. The evidence showed that women use bleaching cream during pregnancy and postnatal for improving their appearance. This is considered a social aspect, as women, mostly from sub-Saharan Africa, feel the need to maintain their normal appearances during pregnancy and postnatal because they are affected by how the society “judges” their appearance. Marital status was found to be associated with LBW, preterm birth and retained placenta. Single mothers were at higher risk of adverse outcomes due to lack of socioeconomic support, both financial and emotional support, from their husbands and family [34] 

### 3.2. Physical Environmental Factors (Table 2)

In a study spanning 23 sub-Saharan African countries, it has been reported that exposure to environmental tobacco smoke (ETS) by mothers during pregnancy causes low birth weight in new-borns, particularly of the male gender [15]. Environmental toxicants (chromium [Cr]) exposure is associated with pre-eclampsia [43]. Exposure to pollutant cooking fuel is associated with LBW [15]. Exposure to household air pollution (HAP) is associated with placental function (placental inflammation, placental hypoxia and thrombotic placental lesions) [44,45]. Exposure to these fuels affects placental function. In these studies, firewood and kerosene were considered fuels that caused household air pollution. Exposure to heat or the sun is associated with LBW [24]. According to this evidence, mothers who stood in the sun or near a fire source for a long time are at high risk of LBW, although there is no explanation for this association. 

**Figure 3:**
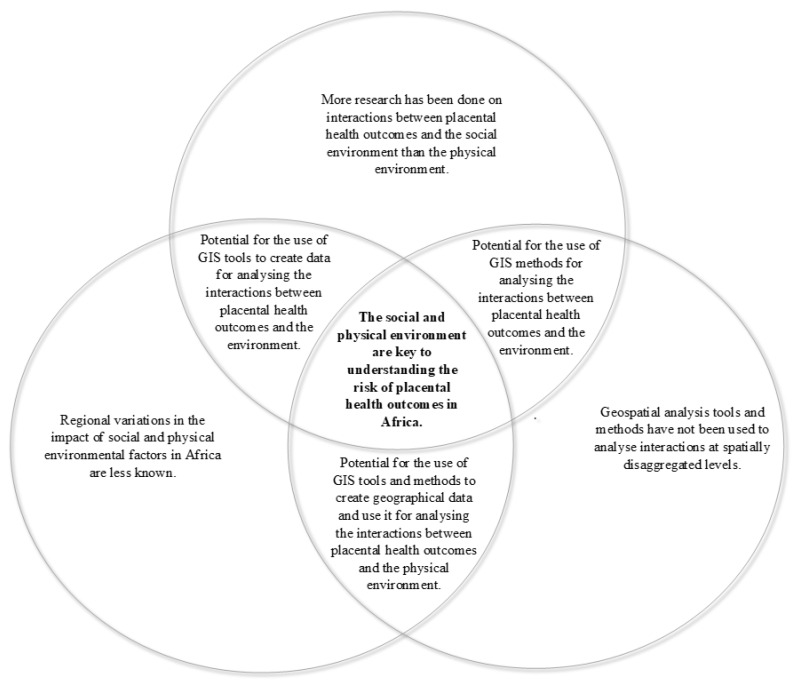
Key messages form the scoping review. GIS: Geographical Information Systems.

The key messages from this scoping review are highlighted in Figure 3.

## 4. Discussion

This scoping review identified current evidence related to physical and social environmental effects on pregnancy events related to placental disorders in Africa. The review has revealed the important role played by the social and physical environment in stratifying risk of placental health outcomes. A lot of research has been done to investigate sociodemographic factors and their influence on placental health outcomes.

The physical environment has been found to affect obstetric outcomes, even though not much has been reported, and its interaction with placental health outcomes, as compared to the social environment. More studies have investigated the influence of the social environment, especially at an individual level, on placental function compared to the physical environment. More has been done on this subject in the more developed parts of the world than in Africa. The review revealed that not much is known about the leading outcomes associated with the placental function, that is, pre-eclampsia (hypertension), IUGR and stillbirths.

The existing evidence used the mother’s season of birth as the measure of her own early life environment. Upon validation of this measure and investigating the association between maternal birth season and placental function, there is a need to determine if effects of foetal undernourishment of mothers can be countered by proper nourishment during pregnancy. Knowledge of the mother’s foetal nourishment will play a big role in determining her baby’s risk, thus informing decision makers on the required nourishment during her pregnancy. This determinant is crucial because if not addressed, the cycle of underweight infants will not be broken until a generation is properly nourished.

The association between sex of the baby and LBW leads to the question, “Can then the population structure of a community be used to determine the risk of LBW, hence inform policy makers or researchers on which areas require more interventions to reduce risk of LBW?” More research needs to be done to determine the association between the sex of the baby and placental disorders as such knowledge will play a role in determining risk of LBW in babies.

Although existing evidence showed that booking for pregnancy care at health facility improves outcomes, there still exists the question of whether early booking will play a bigger role in improving these maternal outcomes. Women’s autonomy plays a big role in their health care seeking behaviour [58]. Decision and financial autonomy are factors that may play a big role in determining pregnant women’s health seeking behaviour. As this is an issue in the patriarchal societies in Africa, there is a need to understand the extent to which autonomy affects maternal health, specifically placental function.

From the evidence that exists, conflicting results, on the nature of the relationships between the environment and placental health outcomes, exist for some factors across the studies. More evidence is needed to determine the relationships between the environment and placental function. Most of the existing literature showed that these studies were done in referral facilities where cases are critical. Most, if not all women who were referred to the study facilities were in critical conditions. There is a need to investigate the interactions from within the communities.

The regional variations in the impact of identified environmental factors on outcomes related to placental disorders in Africa is less clear. Only one study investigated the effect of place of delivery as a function of distance, and only in relation to LBW. Less is known about the influence of geographical access to care on outcomes related to placental disorders. Travel time (dependent on mode of transport and road infrastructure) is a known measure for physical access to care that models risk better than distance [59]. The seasonal elements also affect the travel times, as precipitation and floods tend to increase the time to access care. A model that took all these factors into account was derived to determine the variations in travel times across seasons and to be used as a tool to inform risk due to geographical access [59]. Using such a model to investigate the effect of travel time and seasonal variations on outcomes related to placental disorders across different societies in the country and across different regions in Africa will help understand the travel risks and how they differ in and across countries.

There is great potential for the use of Geographical Information Systems (GIS) and Geographical Information Science (GISc) tools and methods in collecting and manipulating data for use as measures for social and physical environmental indicators. There are studies that have used geographical methods to measure social indicators, for example using walking distance to major roads as a measure for a community’s isolation [60] and access and proximity to liquor outlets as a measure for alcohol consumption during pregnancy [61,62]. These measures are created using GIS tools and methods, where both collection and manipulation of data is done using GIS technology. Geospatial analysis methods will also play a critical role in the analysis of these interactions at a spatially disaggregated level. Geographically local regression models have the potential of revealing the non-stationarity of the interactions between the environment and the placental health outcomes [60]

## 5. Conclusions

More research on the effects of the social and physical environment will expose how the growing industrialisation, globalisation and migration into cities in Africa is affecting changes in maternal health inequalities, increasing the need to investigate how this change is affecting the placental function. This review has shown that SDHs play a role in determining the nature of placental health outcomes in Africa. What is already known, from existing literature, has shown that there is potential for more research on SDHs relating to placental health in Africa, to further understand the extent of the influence the environment (social and physical) has on placental health. 

## Figures and Tables

**Table 1:** Sample of the search terms used.

Theme	Keywords
Socio-economic or demographic exposures	Social factors, socio-cultural, socio-economic, education, maternal age, parity, antenatal care visits, location, wealth index, nutrition, diet, educational level, sex of baby, geographical location, ethnic background, cultural beliefs.
Physical/environmental exposures	Environmental risk factors, environment influences, environmental hazards, neighbourhood conditions, environmental characteristics, air pollution, pollution, environmental exposure, environmental disparities, contaminants, outdoor air pollution, household air pollution,
Outcomes	Low birth weight, pre-eclampsia, stillbirth, foetal growth restriction, placental abnormalities
Settings	All African countries were listed including Africa and Sub-Saharan Africa keywords

**Table 2:** Summary of investigated factors and outcomes.

	Stillbirth	IUGR	Pre-Eclampsia	Low Birth Weight	Placental Abruption	Preterm Birth	Notes
**Social Exposures**							
**Education Level**				0 [23] 0 [26] 0 [21] 0 [46]			The preterm births were associated with complications caused by placental disorders, which are eclampsia, hypertension, placenta praevia, malaria during pregnancy.
Low education level	1 [14] 2 [22]		1 [16]	1 [24] 2 [25] 1 [18]		2 [47]	
High education level			2 [17]	2 [48] 2 [49]			
**Parity**	0 [31]		0 [50]	0 [25] 0 [31] 0 [39] 0 [51]	0 [31]		The effect of parity changes with different types of stillbirths, which is macerated and recent stillbirths [52]. Compared to pauciparous women, more grand multiparas are of low socioeconomic status and less of them practice prenatal care utilisation [35].
Grand multiparity/multiparity	1 [14] 1 [52] 1 [27] 1 [22]		1 [16] 1 [53]	1 [21] 1 [24]	1 [28] 1 [32] 1 [35] 1 [24]		
Lower parity/primiparity	1 [52] 1 [22]		1 [16]	1 [54] 1 [34]		1 [54]	
**Booking Status**							
Not booking	1 [14]						
**Maternal Age**	0 [22]			0 [25] 0 [46] 0 [51]			Authors speculate that higher education weakens the negative effect higher maternal age has on birth weight [26]. Young and advanced age had a negative effect compared to middle age
Older age	1 [14] 1 [52]		1 [16]	2 [26] 2 [48] 1 [39] 1 [40] 1 [49]			
Younger age	1 [14] 1 [27]			1 [24] 1 [23] 1 [54] 1 [21] 1 [55]		1 [54] 1 [47]	
**Residence**							In the Gambia, primary health care (PHC) villages have village health workers and resident traditional birth attendants because they have a population of more than 400 inhabitants [54].
Primary health care village				1 [54]		1 [54]	
Non-Primary Health Care Village							
**Rural/Urban Residency**				0 [25] 0 [49]			
Rural				1 [30] 1 [46] 2 [48]		1 [47]	
**Wealth Index**				0 [25] 0 [49]			
Higher				2 [48]			
Lower				1 [30]			
**Maternal employment**							The protective or risk effect of the woman being employed on placental health outcomes depends on the type of work the woman does, that is, whether it is labour-intensive employment or not.
Employed		1 [56]		1 [21] 2 [48]		1 [47]	
Not employed	2 [27]				1 [28]		
**Maternal Season of Birth**							Adjusting for offspring season of birth does not alter the magnitude of change in birth weight of offspring of women born in different birth seasons [38]. This could mean that the effects of a woman’s bad nutrition during their season of birth may affect her baby’s birthweight, despite the baby receiving good nutrition.
Hunger season				1 [38]			
**Paternal Season of Birth**				0 [38]			
**Prenatal Care**				0 [25] 0 [51]			Physical access is not a problem in Botswana; hence, the rate of antenatal care seeking is good [23].
Seeking		2 [56]		2 [24] 2 [23] 2 [46]			
Not seeking/less visits			1 [46]	1 [34] 1 [18]		1 [47]	
**Nutrition**						2 [47]	Cultural beliefs in Northern Ghana affect women’s weight gain as some beliefs state that if you gain weight, the baby will gain weight causing a difficult delivery or leading to a caesarean section birth [46]. Therefore, pregnant women may deprive themselves of food fearing a difficult delivery. There is a claim that a nutritious drink called “mahewu” (a traditional drink in Zimbabwe) has protective qualities if drunk during pregnancy [47]. Authors suggest that studies are needed to investigate whether nutrition affects the development of the placenta or the nutrition of the foetus [47].
Good nutrition							
No nutrition counselling				1 [49]			
**Marital Status**				0 [21] 0 [49]			Being a married teenager is protective against having a low birth weight (LBW) baby compared to being an unmarried teenager [23]. This effect is not as strong with older women. Married women get family support even from the extend family members [26]. Different socio-cultural practices relating to marriages could be contributing to different nature of relationship between birth weight and marital status in different regions of Ghana [26].
Married				2 [40]	1 [28]		
Not married				1 [23] 1 [29] 1 [26] 1 [18]		1 [47]	
**Type of Union**							
Polygamous							
Monogamous				2 [40]			
**Drinking and Smoking**				1 [24]	0 [28]	1 [47]	Authors found no association between smoking and placental abruption, which they explained as being the result of Nigerien women not having tendencies of smoking [28].
**Socioeconomic Status**				0 [46]			
Higher				2 [26] 2 [21]			
**Use of Skin Lightening Cream**				1 [42]			Placental weight of users of skin lightening cream is significantly lower [42].
**Exposure to Heat or Sun**				1 [24]			Long exposure to the sun and fire during work increases risk of LBW [24].
**Sex of Baby**				0 [54] 0 [51] 0 [49]			The geographical and ethnical differences between the northern [46] and southern [21] regions of Ghana may explain the observed differences in the relationships with LBW.
Boy							
Girl				1 [23] 1 [26] 2 [21] 1 [46] 1 [48]			
**Season**							Supplementation has a greater effect on improving the pregnant woman’s outcomes during the hunger season [57], reducing the odds of having a low birth weight child.
Hunger season				1 [57]			
**Labour Work**				1 [30]			
**Unstable Income**				1 [29]			
**Unplanned Pregnancy**				1 [29]			
**Place of Delivery**							The prevalence of LBW for deliveries attended by traditional birth attendants was lower than that in institutions [40].
**Season**							
Rain season					1 [28]		Increased incidence of placental abruption during the rainy season could be due to the intense field work the women are subjected to [28].
**Ethnicity**				0 [26] 0 [49]	0 [28]		The socio-cultural practices of the major ethnic groups in the study area of the upper eastern region of Ghana are not that different [26], which may have influenced the findings of no association between ethnicity and LBW. Authors recommend a further investigation of the variations of sex of the baby as risk factor of LBW in the northern and southern regions of Ghana [26].
**Religion**				0 [26] 0 [49]			The assumption made by the authors, to explain the nonexistence of an association, is that it is considered taboo in Ghana for women to smoke, therefore the possibility of religion playing a role in reducing the habit of smoking by women is reduced, thus exhibiting no relationship with LBW incidence [26].
**Environmental Exposures**							
**Environment Tobacco Smoke**				1 [48]			The risk effect of exposure to environmental tobacco smoke (ETS) increases in male children [48].
**Environmental toxicants**							In the Democratic Republic of Congo (DRC) and South Africa, studies showed that increased exposure to heavy metals (lead, cadmium and chromium) increased the risk of having preeclampsia [43].
Heavy metals		1 [56]	1 [43]				
**Kitchen location**							
In-house kitchen				1 [48]			
**Household Air Pollution**							
**Pollutant Cooking Fuel**				1 [48]			
**Diet (Vine and Root Vegetables)**				2 [39]			Authors asked the question, “Is it the low cadmium in soil or the inability of the vegetables to absorb it that give home grown vine and root vegetables a protective effect against adverse birth outcomes?”

Note: 1 = Risk, 2 = Protective, 0 = No association.
